# Perceived AI empowerment and threat in relation to university students' dependence on GenAI: the indirect role of trust in AI

**DOI:** 10.3389/fpsyg.2026.1940317

**Published:** 2026-09-23

**Authors:** Ke Zhu, Yuwen Ma, Jin Zhang, Jinhai Liu

**Affiliations:** 1 Faculty of Education, Henan Normal University, Xinxiang, China; 2 Henan Collaborative Innovation Center for Intelligent Education, Henan Normal University, Xinxiang, China; 3 School of Mathematics and Statistics, Henan Normal University, Xinxiang, China

**Keywords:** AI dependence, cognitive appraisal theory, generative artificial intelligence, perceived AI empowerment, perceived AI threat, trust in AI

## Abstract

**Introduction:**

The widespread adoption of generative artificial intelligence (GenAI) in higher education is transforming students' learning practices, yet the psychological factors and associational processes underlying students' dependence on GenAI remain poorly understood. Drawing on cognitive appraisal theory and trust theory, this study proposed a dual-appraisal model to examine how perceived AI empowerment and perceived AI threat are associated with university students' dependence on GenAI, both directly and indirectly through trust in AI.

**Methods:**

A cross-sectional survey was conducted with 360 university students in China who had prior experience using GenAI. The measurement model and hypothesized structural relations were examined using SPSS 24.0 and Mplus 8.3. Indirect associations were evaluated using bootstrap confidence intervals based on 5,000 resamples.

**Results:**

Perceived AI empowerment was positively associated with trust in AI and dependence on GenAI, whereas perceived AI threat was negatively associated with both variables. Trust in AI was positively associated with GenAI dependence and statistically accounted for part of the associations between both cognitive appraisals and dependence. Significant direct and indirect associations were observed in both appraisal pathways.

**Conclusion:**

The findings support a dual-appraisal perspective on university students' dependence on GenAI, suggesting that perceived capability-enhancing benefits and perceived risks are associated with reliance on GenAI through distinct appraisal pathways involving trust. Students who perceived GenAI as more empowering tended to report greater trust and dependence, whereas those who perceived greater AI-related threat reported lower trust and dependence. Trust accounted for significant indirect associations between both forms of appraisal and GenAI dependence. These findings extend research on technology acceptance by highlighting trust as a potential psychological pathway linking students' appraisals of GenAI with their reported reliance on the technology.

## 1 Introduction

Generative artificial intelligence (GenAI) has become an increasingly prominent part of university students' everyday media environment. Students use large language model-based systems to search for information, generate ideas, revise written work, obtain feedback, and solve academic problems (Chan and Hu, 2023). Unlike conventional digital tools that mainly retrieve or organize existing content, GenAI generates individualized responses through continuous interaction, allowing it to function as an interactive cognitive medium embedded in students' learning routines (Schutte and Li, 2025). Although these affordances may improve efficiency and support task completion (Kong et al., 2025), they also raise a key question: why do some students use AI as a supplementary resource, whereas others increasingly rely on it for cognitive and academic activities? More broadly, research on technology-supported learning environments suggests that students' learning experiences and outcomes depend not only on technological affordances but also on the psychological processes that shape how learners engage with these environments (Zhang et al., 2026).

Answering this question requires distinguishing AI use from GenAI dependence. Recent research has identified GenAI dependence among college students as an emerging concern, highlighting the need to understand how reliance on GenAI may influence students' cognitive activities, decision-making, and learning autonomy (He et al., 2026). Frequent use does not necessarily indicate dependence, because students may continue to verify AI outputs, exercise independent judgment, and retain control over academic decisions (Nazaretsky et al., 2025). GenAI dependence instead refers to a more persistent tendency to delegate information processing, idea generation, problem solving, and task completion to an AI system (Wang T. et al., 2026). AI dependence should therefore be distinguished from simple usage frequency. Frequent interaction with GenAI does not necessarily indicate dependence, as students may use GenAI as a supplementary resource while maintaining independent judgment, verification, and decision authority. Beyond the distinction between AI use and AI dependence, recent perspectives on human-AI interaction emphasize the role of AI augmentation. AI augmentation refers to the use of AI systems to enhance human capabilities while preserving users' cognitive agency, judgment, and decision-making authority. In educational settings, students may use GenAI to extend their learning capacity, improve efficiency, and obtain support for complex tasks without necessarily becoming dependent on GenAI (Daniel et al., 2025). Therefore, frequency of AI use alone cannot determine whether students are engaging in productive augmentation or developing stronger dependence on GenAI (Qian, 2025). In contrast, AI dependence reflects the extent to which students rely on GenAI for cognitive and task-related activities and experience greater difficulty performing these activities without AI assistance. Such reliance may exist along a continuum, ranging from functional reliance, in which AI supports task completion while users retain cognitive control, to potentially problematic dependence, in which users increasingly relinquish independent verification and decision making. This distinction is important because GenAI may support learning when it complements students' reasoning, but excessive or inappropriate reliance may weaken autonomous learning, critical evaluation, and sustained cognitive engagement (Hao et al., 2026). Existing research has mainly explained GenAI adoption and continued use through perceived usefulness, performance benefits, ease of use, and task–technology fit (Khoso et al., 2025; Liu and Wei, 2026). These perspectives clarify why individuals choose to use digital technologies, but they offer a less complete explanation of why repeated interaction may develop into cognitive reliance. However, because GenAI-generated information may contain inaccuracies, biases, or contextually inappropriate suggestions, students' reliance on GenAI also depends on their ability and willingness to evaluate and verify AI outputs.

A fuller account of GenAI dependence requires attention to how students appraise both the benefits and risks of GenAI. Students may regard AI as empowering because it enhances access to information, improves task performance, and supports problem solving. At the same time, they may perceive it as threatening because of concerns about misinformation, privacy, academic integrity, reduced autonomy, and long-term competence development (Wang X. et al., 2026). Cognitive appraisal theory suggests that individuals evaluate external conditions in relation to their goals, resources, control, and potential losses. In this study, perceived AI empowerment refers to the belief that AI enhances personal capability and task performance (Kong et al., 2025; Spreitzer, 1995), whereas perceived AI threat refers to the belief that AI may introduce risks or negatively affect learning and future development (Kieslich et al., 2021). These appraisals are not necessarily mutually exclusive; students may recognize GenAI's functional value while remaining concerned about its consequences.

Appraisals alone may not fully explain students' willingness to rely on GenAI. Trust is likely to be important because GenAI is both interactive and partly opaque: users receive personalized responses but often cannot observe how those responses are generated or fully assess their reliability (Park and Yoon, 2025). Reliance may therefore depend on whether the system is perceived as competent, reliable, and predictable (O'Brien, 2025). Trust theory similarly proposes that reliance is more likely when users hold positive expectations about an agent's ability and dependability (Mayer et al., 1995). Perceived empowerment may strengthen such expectations and be associated with greater AI dependence, whereas perceived threat may weaken trust and discourage reliance (Kalimira and Jazayeri, 2026; Yin, 2025). Trust may therefore represent an important psychological link between students' appraisals of GenAI and their reported dependence, yet this indirect relationship remains insufficiently examined.

Perceived AI empowerment refers to users' sense that AI helps them perform tasks more effectively. As students increasingly integrate GenAI into academic activities, the technology may gradually shift from an occasionally consulted tool to a more routine source of cognitive support (Colombari et al., 2026). Importantly, students' reliance on GenAI may be shaped not only by how frequently they interact with AI systems but also by the quality of these interactions and the psychological evaluations formed during human–AI engagement. Thus, trust and dependence may emerge from students' perceptions of AI usefulness, reliability, and experiential value rather than from repeated exposure alone (Li, 2025). Frequent use is not equivalent to dependence, but repeated interaction may shape users' judgments of AI's capabilities, risks, and reliability. The present study therefore integrates cognitive appraisal theory and trust theory to examine how perceived AI empowerment and perceived AI threat are associated with university students' GenAI dependence, both directly and indirectly through trust in AI. It also explores whether these relationships show different patterns across AI usage-frequency groups. The study addresses the following research questions:

RQ1: How are perceived AI empowerment and perceived AI threat associated with university students' trust in AI and GenAI dependence?
RQ2: Does trust in AI account for significant indirect associations between perceived AI empowerment, perceived AI threat, and GenAI dependence?
RQ3: Do these associations differ across AI usage-frequency groups?

## 2 Literature review and hypotheses

### 2.1 Perceived AI empowerment

Perceived AI empowerment refers to users' sense that AI helps them perform tasks more effectively, work more efficiently, and handle problems that would otherwise be difficult. The concept concerns a subjective experience of increased competence and control, rather than the objective capabilities of the technology itself (Spreitzer, 1995). In the context of GenAI, students may feel empowered when AI helps them process complex information, organize ideas, generate content, or improve the quality of their work (Kim et al., 2026). Previous research has shown that AI can support judgment by providing information processing, analysis, and recommendations in complex tasks (Jarrahi, 2018). In higher education, it is also used for learning support, assessment feedback, and personalized instruction (Zawacki-Richter et al., 2019). Beyond supporting task completion and efficiency, AI-assisted learning may also contribute to students' motivation, engagement, creativity, and learning outcomes through multiple pathways. Therefore, students' perceptions of AI empowerment may involve not only instrumental benefits but also broader psychological and learning-related experiences (Chen, 2024). Students who view GenAI as useful, convenient, and supportive of learning are generally more willing to adopt it and incorporate it into their academic activities (Firaina and Sulisworo, 2023). However, perceived AI empowerment should be distinguished from students' general readiness to engage with AI. AI readiness reflects individuals' confidence, preparedness, and perceived capability in using AI technologies, which may influence their learning experiences and outcomes. In contrast, perceived AI empowerment concerns whether students feel that AI enhances their competence, effectiveness, and ability to accomplish tasks. Therefore, AI empowerment represents a psychological appraisal of AI's supportive role rather than merely an individual capability to use AI (Wang and Li, 2024).

Within this framework, perceived AI empowerment may encourage students to integrate GenAI into academic activities. However, whether such integration develops into dependence depends on how students regulate their reliance and maintain cognitive autonomy. Such reliance may remain functional when AI is used as a supplementary resource and students retain independent verification and final decision authority (Klingbeil et al., 2024). However, as reliance becomes stronger, students may increasingly favor AI-generated recommendations, delegate more cognitive work to the system, or become less willing to complete certain tasks independently (Passi and Vorvoreanu, 2022; Zhang et al., 2024). Under these conditions, functional reliance may gradually approach potentially problematic dependence, particularly when independent verification and autonomous judgment are reduced.

Cognitive appraisal theory suggests that people evaluate external resources according to whether those resources help them achieve important goals (Lazarus, 1991). When students believe that AI improves efficiency, extends their capabilities, and makes academic tasks easier to manage, they are likely to view it as a valuable learning resource. This appraisal may increase their willingness to turn to AI in later tasks and to rely on it for activities such as information processing, content generation, and problem solving. On this basis, the following hypothesis is proposed:

***H1:*** *Perceived AI empowerment is positively associated with AI dependence*.

Perceived AI empowerment may also be related to trust in AI. Trust under conditions of uncertainty depends partly on whether an agent is regarded as capable and trustworthy (Mayer et al., 1995). Research on trust in automation and AI similarly shows that perceived competence, reliability, and task performance are important foundations of trust (Glikson and Woolley, 2020). When students experience AI as useful in solving problems, saving time, and improving task performance, they may come to see the system as more capable and dependable. Perceived empowerment is therefore expected to be associated with stronger trust in AI.

***H2:*** *Perceived AI empowerment is positively associated with trust in AI*.

### 2.2 Perceived AI threat

Perceived AI threat refers to users' appraisal of the possible negative consequences of AI use, including concerns about diminished competence, privacy loss, academic misconduct, reduced learning quality, and uncertainty about future development (Pikhart and Al-Obaydi, 2025). It differs from general technology anxiety in that it focuses less on discomfort with technology itself and more on anticipated outcomes of using it. In the GenAI context, students may perceive threat when they believe that AI could weaken independent thinking, blur responsibility, or adversely affect their learning and longer-term development (Nazaretsky et al., 2025; Oc et al., 2025).

Cognitive appraisal theory holds that people interpret external events in relation to their goals, resources, and sense of control. When a technology is seen as a possible source of loss, reduced control, or future harm, it is more likely to evoke defensive or avoidant responses (Folkman, 2013). For university students, concerns about learning passivity, declining critical thinking, privacy breaches, academic misconduct, or future job displacement may shift GenAI from a learning aid to a perceived threat (Sousa and Cardoso, 2025). Previous research likewise shows that students' responses to GenAI are often accompanied by concerns about ethical risks, capability replacement, and uncertain consequences, which may increase hesitation and avoidance (Ivanov et al., 2024; Yin, 2025). From a cognitive appraisal perspective, perceived AI threat represents users' evaluation that AI systems may introduce uncertainty, risks, or negative consequences into learning activities (Folkman, 2013). Such threat perceptions may undermine trust in AI because trust is fundamentally based on expectations regarding system reliability, competence, and predictability (Afroogh et al., 2024). In the context of GenAI, concerns about inaccurate outputs, hallucinations, limited transparency, and insufficient contextual understanding may reduce students' confidence in AI-generated information. When students perceive that AI outputs are less reliable or difficult to evaluate, they may be less willing to place trust in AI systems. Therefore, perceived AI threat is expected to be negatively associated with trust in AI (Cheng et al., 2026).

Perceived threat may first be associated with lower trust in AI. Trust depends partly on positive expectations about a system's competence, reliability, and predictability. These expectations are likely to weaken when users perceive greater risk or uncertainty. Students form trust judgments on the basis of output accuracy, feedback quality, privacy protection, and possible effects on learning (Henderson et al., 2026). When AI is viewed as a threat to autonomy, academic integrity, or future competence, students may become more doubtful about its safety and reliability. Accordingly, the following hypothesis is proposed:

***H3:*** *Perceived AI threat is negatively associated with trust in AI*.

Perceived AI threat may also be associated with lower GenAI dependence. Technology threat avoidance theory suggests that perceived harm can activate protective motives, leading users to reduce exposure, verify information more carefully, or avoid the technology altogether (Liang and Xue, 2009). In academic settings, students who believe that AI may weaken independent judgment or create learning and integrity risks may be less willing to delegate task completion, knowledge evaluation, and problem solving to the system. Consistent with this reasoning, concerns about the risks and adverse consequences of GenAI have been linked to hesitation, resistance, and more cautious use (Cabrera and Neville, 2025). Therefore:

***H4:*** *Perceived AI threat is negatively associated with AI dependence*.

### 2.3 The mediating effect of trust in AI

Trust in AI refers to a psychological state in which users hold positive expectations about an AI system based on judgments of its competence, reliability, predictability, and safety. Unlike general attitudes toward technology or perceived usefulness, trust becomes particularly relevant when users must rely on a system despite limited transparency, uncertain outputs, and potential risks (Shin, 2021). General trust theory suggests that reliance under uncertainty depends partly on positive expectations about an agent's ability and trustworthiness (Mayer et al., 1995). This issue is especially salient for GenAI because students cannot fully observe its algorithmic logic, information sources, or response-generation process. Their judgments of the system's competence and reliability may therefore play an important role in how they subsequently use it (Mitchell, 2025).

Research on automation and AI has consistently shown that trust influences whether users accept system recommendations and rely on them during task performance (Lee and See, 2004). In learning contexts, students who regard AI outputs as accurate, useful, and reasonably consistent may be more willing to accept its suggestions and incorporate it into information seeking, content generation, and problem solving (Zhao et al., 2026). Lower trust, by contrast, is more likely to prompt doubt, verification, and greater distance from the system. Trust in AI should therefore be associated with students' willingness to delegate cognitive and academic activities to GenAI.

***H5:*** *Trust in AI is positively associated with AI dependence*.

Trust may also help explain how students' appraisals of GenAI are associated with dependence. When students perceive AI as improving efficiency, extending their capabilities, and providing valuable academic support, these experiences may strengthen their expectations of the system's competence and reliability (Choung et al., 2023). Stronger trust may, in turn, increase their willingness to accept AI-generated suggestions and incorporate the system into routine learning activities. Perceived AI empowerment may thus be indirectly associated with greater dependence through trust in AI.

The opposite pattern may arise when students view GenAI as a threat. Concerns about diminished autonomy, academic integrity, unreliable outputs, or uncertain long-term consequences may weaken beliefs in the system's safety, reliability, and predictability (Yin, 2025). Reduced trust may then encourage closer verification, less task delegatin, and more cautious engagement with AI (Raees and Papangelis, 2026). Information systems research likewise identifies trust as an important link between users' technology-related beliefs and their subsequent acceptance and use behaviors (Gefen et al., 2003; McKnight et al., 2002). Trust in AI may therefore carry the indirect associations of both empowering and threatening appraisals with GenAI dependence.

***H6a***: *Trust in AI mediates the positive association between perceived AI empowerment and AI dependence*.
***H6b***: *Trust in AI mediates the negative association between perceived AI threat and AI dependence*.

Taken together, these arguments support a dual-path model in which perceived AI empowerment and perceived AI threat are associated with university students' GenAI dependence through both direct paths and indirect paths involving trust in AI. Perceived empowerment is expected to be associated with greater trust and dependence, whereas perceived threat is expected to be associated with lower trust and dependence. The proposed model is presented in Figure 1.

**Figure 1:**
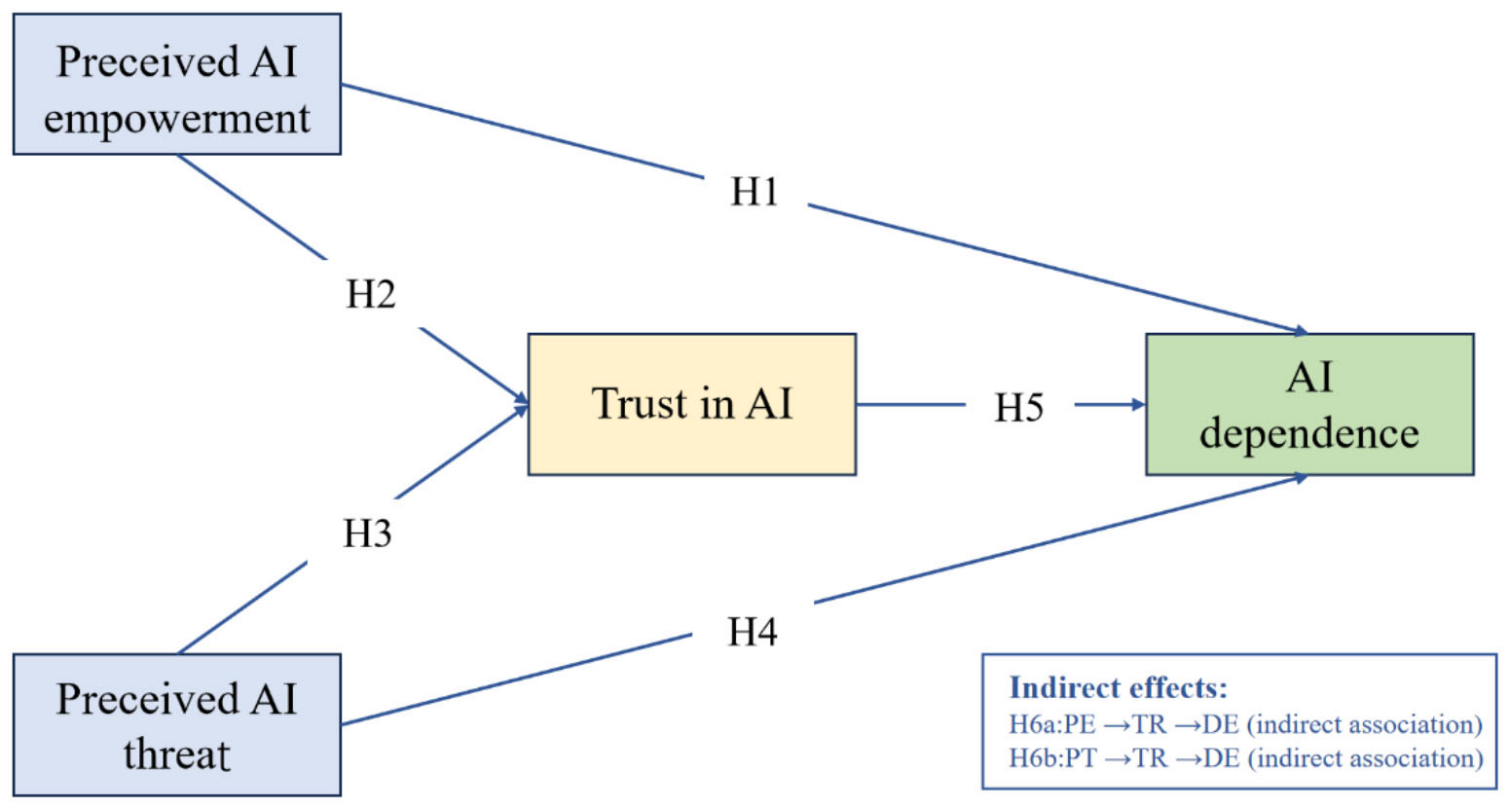
Proposed research model.

## 3 Method

### 3.1 Participants and procedure

This study employed a cross-sectional questionnaire design, targeting Chinese university students with experience using GenAI. The questionnaire was distributed online via the online platform and disseminated through university course groups, learning communities, and social media channels. Participants were recruited using convenience sampling. Prior to completing the questionnaire, all participants were informed about the purpose of the study, the voluntary nature of participation, their right to withdraw at any time, and the confidentiality and anonymity of their responses. Electronic informed consent was obtained from all participants before they proceeded to the survey. The study involved human participants and was conducted in accordance with the Declaration of Helsinki and relevant ethical guidelines. The study protocol was reviewed and approved by the Ethics Committee of the Henan Collaborative Innovation Center for Intelligent Education, Henan Normal University (Approval Code: 2026BS-0523).

Data collection took place from April 24 to May 25, 2026, across universities in multiple provinces and cities including Henan and Hubei. A total of 739 questionnaires were initially collected. To ensure data quality, strict screening procedures were applied prior to formal analysis. Excluded responses included those with completion times shorter than the preset minimum threshold (1 min), consecutive identical answers on more than 10 questions, or logical inconsistencies. After data cleaning, 360 valid questionnaires were retained for subsequent analysis.

As shown in Table 1, the final sample consisted of 360 GenAI-experienced university students, including 174 males (48.3%) and 186 females (51.7%). Age distribution: 72.8% were under 22 years old, 12.2% aged 22–25, 11.4% aged 26–30, and 3.6% over 30. Grade distribution: 50.8% were freshmen, 25.3% sophomores, 15.0% juniors, and 8.9% seniors or above. Regarding GenAI usage frequency: 73 students (20.3%) reported “rarely using” ( ≤ 1 time per month), 150 (41.7%) reported “sometimes using” (1–3 times per week), and 137 (38.1%) reported “frequently using” (≥4 times per week).

**Table 1:** Demographic characteristics of the study sample.

Variable	Category	*N*	%
Gender	Male	174	48.3
	Female	186	51.7
Age	< 22 years old	262	72.8
	22–25 years old	44	12.2
	26–30 years old	41	11.4
	>30 years old	13	3.6
Grade	Freshman year	183	50.8
	Sophomore year	91	25.3
	Junior year	54	15.0
	Senior year and above	32	8.9
GenAI usage frequency	Few times	73	20.3
	Sometimes	150	41.7
	Frequently	137	38.1

### 3.2 Measures

#### 3.2.1 Perceived AI empowerment

Perceived AI empowerment was measured using seven items adapted from the psychological empowerment framework of Spreitzer (1995) and subsequent research on AI empowerment by Kong and Yang (2025) and Kong et al. (2025). Because the original measures were not developed specifically for university students' use of GenAI in academic contexts, the wording was contextually adapted to refer to GenAI-supported learning, task completion, and problem solving. These adaptations were intended to preserve the underlying meaning of empowerment as an enhanced sense of competence, effectiveness, and capability while making the items relevant to students' actual use of GenAI. For example, general references to AI-supported capability or problem solving were rephrased to refer explicitly to completing academic tasks with GenAI. A sample item was, “*Using AI makes me feel more capable of completing tasks*.” All items were rated on a five-point Likert scale ranging from 1 (“strongly disagree”) to 5 (“strongly agree”), with higher scores indicating greater perceived AI empowerment. In the present sample, Cronbach's α was 0.945.

#### 3.2.2 Perceived AI threat

Perceived AI threat was measured using five items adapted from the Threats of Artificial Intelligence (TAI) framework developed by Kieslich et al. (2021) and the Artificial Intelligence Anxiety Scale (AIAS) developed by Wang and Wang (2022). The original framework was theoretically grounded in research on fear appeals and conceptualized AI threat as users' cognitive appraisal of the potentially adverse consequences of AI rather than emotional fear itself. It also emphasized that threat perceptions depend on both the functions performed by AI and the context in which AI is applied. Because the original measure was developed and validated across general AI application domains rather than specifically in GenAI-supported learning, the wording of the five items used in the present study was adapted to reflect university students' use of GenAI and its potential consequences for learning and task-related activities. The adaptations primarily involved replacing general or domain-specific AI references with GenAI-related educational contexts while retaining the underlying meaning of perceived risk and negative consequences. A sample item was “I am concerned that using AI may lead to unforeseen negative consequences.” All items were rated on a five-point Likert scale ranging from 1 (“strongly disagree”) to 5 (“strongly agree”), with higher scores indicating greater perceived AI threat. In the present sample, Cronbach's α was 0.934.

#### 3.2.3 Trust in AI

Trust in AI was measured with six items adapted from the Trust in Automation Scale (Jian et al., 2000). The item wording was adjusted for the GenAI context. The scale assessed students' positive expectations regarding AI competence, reliability, and the support it could provide. A sample item was, “*I believe that AI can provide valuable support in most situations*.” All items were rated on a five-point Likert scale ranging from 1 (“strongly disagree”) to 5 (“strongly agree”), with higher scores indicating greater trust in AI. In the present sample, Cronbach's α was 0.933.

#### 3.2.4 AI dependence

AI dependence was measured with seven items adapted from prior research on dependence on AI, including the AI dependence framework proposed by Morales-García et al. (2024) and the AI dependence Scale developed by Wu et al. (2026). The items were revised to reflect university students' use of GenAI. The scale assessed the extent to which students relied on GenAI when completing tasks, solving problems, and carrying out related activities. A sample item was, “*I feel that completing tasks becomes more difficult when I do not use AI*.” All items were rated on a five-point Likert scale ranging from 1 (“strongly disagree”) to 5 (“strongly agree”), with higher scores indicating greater GenAI dependence. In the present sample, Cronbach's α was 0.954.

### 3.3 Data analysis

Data were analyzed using SPSS 24.0 and Mplus 8.3. First, SPSS 24.0 was used for data screening, descriptive statistics, and Pearson correlation analysis. Harman's single-factor analysis was used only as an initial diagnostic for common variance. Confirmatory factor analysis (CFA) was then performed in Mplus 8.3 to examine the measurement structure of perceived AI empowerment, perceived AI threat, trust in AI, and GenAI dependence. The hypothesized four-factor model was compared with a single-factor model. Model fit was evaluated using the chi-square statistic (χ^2^), comparative fit index (CFI), Tucker–Lewis index (TLI), root mean square error of approximation (RMSEA), and standardized root mean square residual (SRMR).

After the measurement model was supported, structural equation modeling (SEM) was used to test the hypothesized paths. These included the associations of perceived AI empowerment and perceived AI threat with trust in AI and AI dependence, as well as the association between trust in AI and AI dependence. The indirect effects through trust in AI were tested using bootstrap estimation with 5,000 resamples. Ninety-five percent confidence intervals were reported, and an indirect effect was considered statistically significant when the interval did not include zero. Direct, indirect, and total effects were reported to evaluate the mediating role of trust in AI in the two appraisal pathways. All path coefficients are presented as standardized STDYX estimates.

Finally, a multigroup structural equation model was used to explore the stability of the structural relationships across low-, moderate-, and high-frequency AI use groups. An unconstrained model was compared with models in which individual structural paths were constrained to be equal across groups. Group-specific path coefficients were also reported to describe the pattern of associations within each usage-frequency group. When equality constraints did not produce a significant deterioration in model fit, differences in group-specific coefficients were interpreted only as exploratory and descriptive.

## 4 Results

### 4.1 Common method bias tests

Because all core variables were measured using self-report questionnaires at a single time point, potential common method bias was assessed before the main analyses. Harman's single-factor test identified four factors with eigenvalues greater than 1, and the first factor accounted for 47.190% of the total variance. Given the relatively large proportion explained by the first factor, a confirmatory factor analysis was conducted to compare the fit of the single-factor model with that of the hypothesized four-factor model.

As shown in Table 2, the four-factor model showed good fit to the data (χ^2^ = 381.950, df = 269, CFI = 0.986, TLI = 0.984, RMSEA = 0.034, SRMR = 0.029), whereas the single-factor model fit the data poorly (χ^2^ = 3735.159, df = 275, CFI = 0.492, TLI = 0.446, RMSEA = 0.187, SRMR = 0.177). These results indicate that a single latent factor did not adequately account for the covariance among the observed variables, providing no evidence that the observed relationships were primarily driven by one common method factor. Nevertheless, because the study relied on cross-sectional, single-source self-report data, common method bias cannot be ruled out entirely.

**Table 2:** Comparison of the four-factor and single-factor measurement models.

Model	χ^2^	df	CFI	TLI	SRMR	RMSEA
Four-factor model	381.950	269	0.986	0.984	0.029	0.034
Single-factor model	3735.159	275	0.492	0.446	0.177	0.187

### 4.2 Descriptive statistics and correlations

Table 3 presents the descriptive statistics and Pearson correlations among the study variables. Perceived AI empowerment (PE) was positively correlated with trust in AI (TR; *r* = 0.532, *p* < 0.01) and AI dependence (DE; *r* = 0.446, *p* < 0.01). Perceived AI threat (PT), by contrast, was negatively correlated with trust in AI (*r* = −0.288, *p* < 0.01) and AI dependence (*r* = −0.316, *p* < 0.01). Trust in AI was also positively correlated with AI dependence (*r* = 0.602, *p* < 0.01). The correlation between perceived AI empowerment and perceived AI threat was weak and non-significant (*r* = 0.030), suggesting that the two appraisals were relatively independent in the present sample.

**Table 3:** Descriptive statistics and correlations.

	PE	PT	TR	DE
PE	1			
PT	0.030	1		
TR	0.532^**^	−0.288^**^	1	
DE	0.446^**^	−0.316^**^	0.602^**^	1
*Mean*	3.038	2.936	3.139	3.169
*SD*	1.145	1.212	1.158	1.220

^**^p<*0.01*, PE, perceived AI empowerment; PT, perceived AI threat; TR, trust in AI; DE, AI dependence.

### 4.3 Measurement model assessment

Table 4 presents the reliability and convergent validity results for the measurement model. The standardized factor loadings ranged from 0.804 to 0.883. Cronbach's α values ranged from 0.933 to 0.954, and composite reliability (CR) values ranged from 0.933 to 0.954, indicating high internal consistency across the four constructs. The average variance extracted (AVE) values ranged from 0.699 to 0.749, providing support for convergent validity.

**Table 4:** Measurement model assessment.

Constructs	Items	Standardized factor loadings	Cronbach's α	CR	AVE
PE	PE1	0.822	0.945	0.945	0.710
	PE2	0.823			
	PE3	0.851			
	PE4	0.883			
	PE5	0.843			
	PE6	0.840			
	PE7	0.835			
PT	PT1	0.868	0.934	0.934	0.739
	PT2	0.861			
	PT3	0.854			
	PT4	0.854			
	PT5	0.860			
TR	TR1	0.804	0.933	0.933	0.699
	TR2	0.844			
	TR3	0.830			
	TR4	0.859			
	TR5	0.842			
	TR6	0.838			
DE	DE1	0.860	0.954	0.954	0.749
	DE2	0.871			
	DE3	0.850			
	DE4	0.861			
	DE5	0.860			
	DE6	0.875			
	DE7	0.880			

PE, perceived AI empowerment; PT, perceived AI threat, TR, trust in AI; DE, AI dependence.

Discriminant validity was further assessed using the Fornell–Larcker criterion and the heterotrait–monotrait ratio (HTMT). As shown in Table 5, the square root of the AVE for each construct was greater than its correlations with the other constructs. Table 6 also shows that all HTMT values were below 0.85, with the highest value being 0.683. Taken together, these results support the internal consistency, convergent validity, and discriminant validity of the measurement model.

**Table 5:** Fornell–Larcker criterion.

Constructs	DE	PE	PT	TR
DE	0.865			
PE	0.446	0.843		
PT	−0.318	0.027	0.859	
TR	0.603	0.532	−0.291	0.836

PE, perceived AI empowerment; PT, perceived AI threat; TR, trust in AI; DE, AI dependence.

**Table 6:** Heterotrait–monotrait ratio (HTMT).

Constructs	DE	PE	PT	TR
DE				
PE	0.470			
PT	0.335	0.054		
TR	0.683	0.566	0.308	

PE, perceived AI empowerment; PT, perceived AI threat; TR, trust in AI; DE, AI dependence.

Although perceived AI empowerment and trust in AI were moderately correlated (*r* = 0.532), the discriminant-validity results supported their empirical distinctness. The square roots of AVE for perceived AI empowerment (0.843) and trust in AI (0.836) exceeded the PE-TR correlation, and the PE-TR HTMT value was 0.566, below 0.85. These findings indicate that empowerment reflects a capability-enhancing appraisal of AI, whereas trust reflects positive expectations regarding AI competence, reliability, and dependability.

### 4.4 Structural model and explained variance

The hypothesized structural component was just identified at the latent-variable level. Accordingly, its global fit indices were identical to those of the four-factor CFA and are not interpreted as independent evidence for the hypothesized path directions. Evaluation therefore focuses on the standardized coefficients and bootstrap confidence intervals reported in Table 7 and Figure 2, together with explained variance. Perceived AI empowerment (PE) and perceived AI threat (PT) jointly explained 42.7% of the variance in trust in AI (TR; R^2^ = 0.427). Together, perceived AI empowerment, perceived AI threat, and trust in AI explained 45.9% of the variance in AI dependence (DE; R^2^ = 0.459). Overall, the model showed considerable explanatory power for both trust in AI and AI dependence.

**Table 7:** Structural model and explained variance.

Path	VIF	*f* ^2^	*R^2^*
PE → TR	1.001	0.469	0.427
PT → TR	1.001	0.150	
PE → DE	1.470	0.060	0.459
PT → DE	1.151	0.060	
TR → DE	1.605	0.194	

PE, perceived AI empowerment; PT, perceived AI threat; TR; trust in AI; DE, AI dependence.

**Figure 2:**
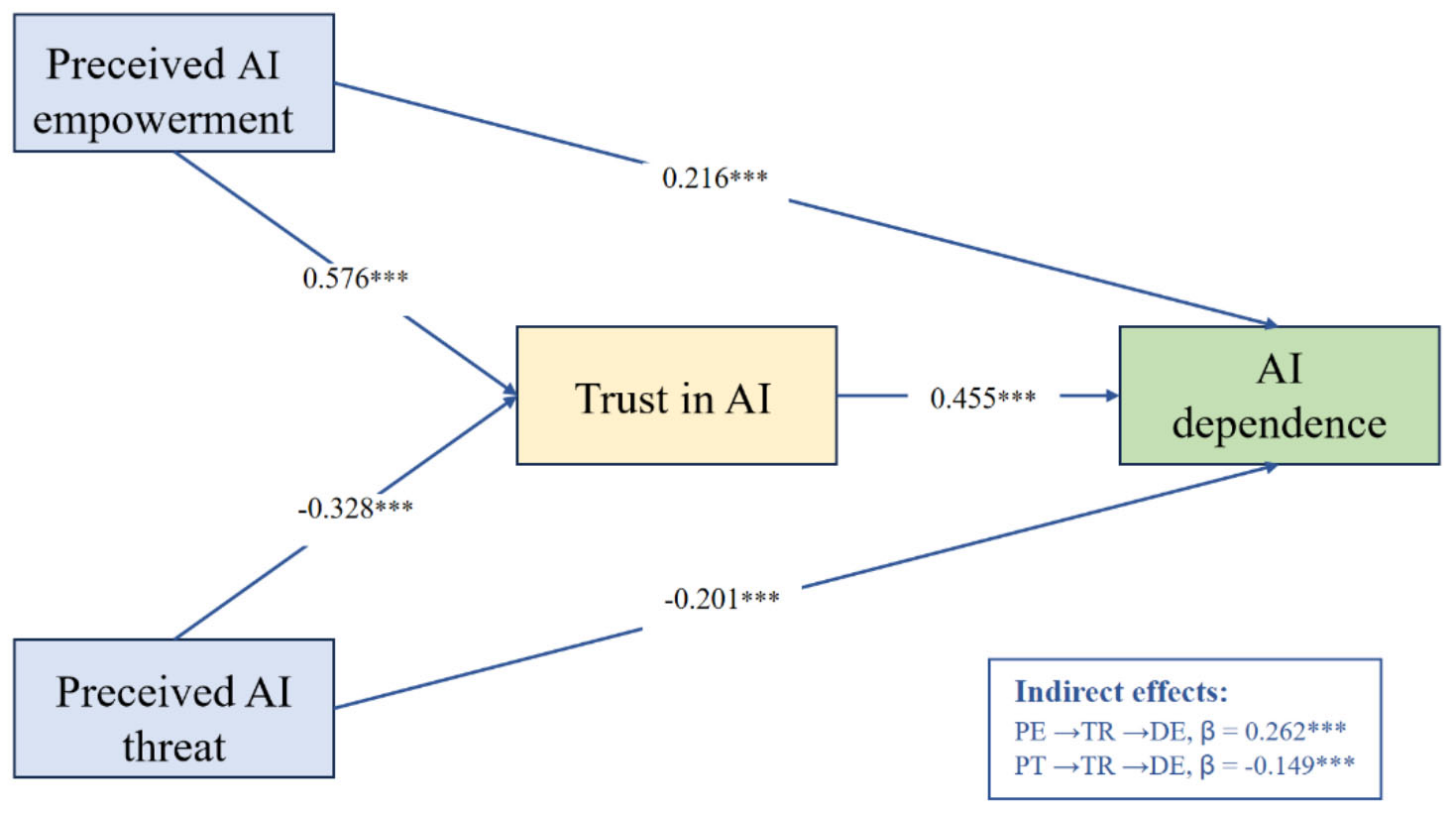
Standardized structural path coefficients.

### 4.5 Hypothesis testing and mediation analysis

The structural path and mediation results are presented in Table 8 and Figure 2. Perceived AI empowerment (PE) was positively associated with trust in AI (TR; β = 0.576, 95% CI [0.493, 0.653]), whereas perceived AI threat (PT) was negatively associated with trust in AI (β = −0.328, 95% CI [−0.420, −0.235]). Trust in AI was positively associated with AI dependence (DE; β = 0.455, 95% CI [0.326, 0.581]). Perceived AI empowerment also showed a significant positive direct association with AI dependence (β = 0.216, SE = 0.059, 95% CI [0.095, 0.328]), while perceived AI threat showed a significant negative direct association with AI dependence (β = −0.201, SE = 0.052, 95% CI [−0.305, −0.099]). Thus, H1–H5 were supported.

**Table 8:** Direct, indirect, and total associations.

Path		Effect	SE	95% CI		Ratio
				Lower	Upper	
Total effect	PE → DE	0.478	0.041	0.393	0.552	
	PT → DE	−0.351	0.046	−0.442	−0.263	
Direct effect	PE → DE	0.216	0.059	0.095	0.328	
	PT → DE	−0.201	0.052	−0.305	−0.099	
	TR → DE	0.455	0.064	0.326	0.581	
Component	PE → TR	0.576	0.042	0.493	0.653	
	PT → TR	−0.328	0.048	−0.420	−0.235	
	TR → DE	0.455	0.064	0.326	0.581	
Total indirect effect	PE → TR → DE	0.262	0.044	0.182	0.356	0.548
	PT → TR → DE	−0.149	0.033	−0.223	−0.094	0.426

PE, perceived AI empowerment; PT, perceived AI threat; TR, trust in AI; DE, AI dependence, Ratio represents the proportion of the total effect mediated by trust in AI; Effect and SE are standardized STDYX estimates.

The total effect of perceived AI empowerment on AI dependence was positive and significant (β = 0.478, SE = 0.041, 95% CI [0.393, 0.552]). The total effect of perceived AI threat on AI dependence was negative and significant (β = −0.351, SE = 0.046, 95% CI [−0.442, −0.263]).

The mediation analysis showed a significant positive indirect association between perceived AI empowerment and AI dependence through trust in AI *(*β = 0.262, SE = 0.044, 95% CI [0.182, 0.356]), accounting for 54.8% of the total effect. Perceived AI threat also showed a significant negative indirect association with AI dependence through trust in AI (β = −0.149, SE = 0.033, 95% CI [−0.223, −0.094]), accounting for 42.6% of the total effect. Because neither confidence interval included zero, H6a and H6b were supported. The direct and indirect effects were significant in both pathways, indicating partial mediation.

### 4.6 Exploratory group comparisons by GenAI usage frequency

This supplementary analysis extends the full-sample findings by mapping how the focal associations are patterned among low-, moderate-, and high-frequency GenAI users. Presenting the group-specific standardized coefficients side by side provides a concise profile of each association's direction, relative magnitude, and consistency across different levels of AI-use experience. The analysis therefore adds practical descriptive value: it identifies common associations that appear robust across usage contexts, highlights numerical gradients that may warrant replication, and helps specify priorities for subsequent cross-group research.

Table 9 presents the standardized coefficients, standard errors, and *p* values within each usage-frequency group. The empowerment-to-trust and threat-to-trust associations showed the same direction across all three groups, whereas the coefficients for the direct associations with AI dependence varied numerically across groups.

**Table 9:** Exploratory association patterns by GenAI usage frequency.

Path	Low-frequency users			Medium-frequency users			High-frequency users		
	β	SE	*p*	β	SE	*P*	β	SE	*p*
PE-TR	0.582	0.087	< 0.001	0.539	0.061	< 0.001	0.607	0.058	< 0.001
PT-TR	−0.369	0.099	< 0.001	−0.341	0.068	< 0.001	−0.297	0.069	< 0.001
PE-DE	0.350	0.133	0.008	0.216	0.076	0.005	0.119	0.091	0.188
PT-DE	−0.115	0.120	0.339	−0.337	0.066	< 0.001	−0.093	0.074	0.213
TR-DE	0.287	0.141	0.042	0.435	0.078	< 0.001	0.593	0.088	< 0.001

The profiles reveal several notable numerical patterns. The trust-to-dependence coefficient increased from 0.287 in the low-frequency group to 0.435 in the moderate-frequency group and 0.593 in the high-frequency group. The direct empowerment-to-dependence coefficient was 0.350, 0.216, and 0.119, *respectively*, whereas the threat-to-dependence coefficient was numerically most negative in the moderate-frequency group. These patterns are presented as descriptive observations rather than evidence of confirmed group differences. Because formal cross-group difference tests and measurement invariance analyses were not conducted, the observed variations should be interpreted cautiously. Future research with larger and more balanced samples should examine whether AI-use experience systematically influences the relationships among appraisal, trust, and dependence.

## 5 Discussion

Drawing on cognitive appraisal theory and trust theory, this study examined the relationships among perceived AI empowerment, perceived AI threat, trust in AI, and AI dependence among university students. Perceived AI empowerment was positively associated with both trust in AI and AI dependence, whereas perceived AI threat was negatively associated with both outcomes. Trust in AI was also positively associated with AI dependence and accounted for significant indirect associations between the two appraisals and dependence. These findings should be interpreted as associations rather than evidence of causal effects. They suggest that students' reported reliance on GenAI is related not only to access or convenience, but also to how they interpret its benefits and risks and to the trust judgments that accompany those interpretations (Garcia, 2026).

Perceived AI empowerment and perceived AI threat showed associations in opposite directions. Consistent with previous research on the educational benefits and risks of GenAI (Arowosegbe et al., 2024), students who viewed AI as improving efficiency, extending task-related capabilities, and reducing cognitive effort tended to report greater trust and dependence (Bu et al., 2026). By contrast, students who were concerned that AI might weaken independent thinking, increase academic risks, or produce uncontrollable consequences reported lower trust and dependence (Al-Emran et al., 2025). The positive association between perceived AI empowerment and AI dependence may reflect different forms of cognitive delegation (Darvishi et al., 2024). When students perceive GenAI as an empowering resource, delegating certain cognitive activities to AI may represent an efficiency-oriented strategy in which AI assists with information processing, idea generation, or task-related support while students retain responsibility for evaluation and decision making. In this case, reliance on GenAI may reflect productive integration of AI into learning practices rather than a loss of cognitive autonomy. In contrast, delegation may have different psychological implications when students perceive GenAI as threatening. Under such circumstances, reliance on AI may not necessarily reflect confidence in AI capabilities, but could instead be associated with uncertainty, lower perceived self-efficacy, or a greater tendency to avoid cognitively demanding tasks (Zhang and Xu, 2025). Therefore, similar forms of reported reliance on GenAI may carry different psychological meanings depending on how students appraise the technology (Wang G. et al., 2026). Notably, the correlation between perceived empowerment and perceived threat was weak and non-significant (*r* = 0.030). Positive and negative appraisals may therefore not represent opposite ends of a single continuum (Hitsuwari and Takano, 2025). Students may recognize the capability-enhancing value of GenAI while remaining concerned about its potential effects. AI dependence is thus better understood as being associated with multiple, coexisting appraisals rather than with a simple orientation of acceptance or rejection (Arce et al., 2025; Zhang et al., 2024).

Trust in AI accounted for significant indirect associations in both appraisal pathways. Perceived empowerment was indirectly associated with stronger AI dependence through higher trust, whereas perceived threat was indirectly associated with lower dependence through reduced trust (Wang and Tian, 2026). These findings suggest that trust may represent an important psychological pathway linking students' appraisals of GenAI with their reported reliance on the technology. According to trust theory, reliance on an agent is more likely when individuals perceive that the agent possesses sufficient ability, reliability, and predictability to support desired outcomes (Mayer et al., 1995). This pattern aligns with research identifying perceived competence, reliability, and performance as important foundations of trust in AI (Glikson and Woolley, 2020; Wang et al., 2024). Because students cannot always verify the sources, reasoning processes, or accuracy of GenAI outputs, their willingness to accept AI-generated suggestions may depend partly on whether they regard the system as capable and reliable (Hu et al., 2026). Therefore, perceived AI empowerment may be associated with greater trust insofar as students view AI as competent and supportive, whereas perceived AI threat may be associated with lower trust because it involves greater uncertainty about AI reliability and potential harm. Perceived empowerment may therefore reflect more than a judgment that AI is useful; it may also co-occur with stronger confidence in the system's competence and supportive value, which in turn is associated with greater reliance (Zhu et al., 2026). Perceived threat, in contrast, may heighten concerns about uncertainty and possible harm, and such concerns may be associated with lower trust and weaker reported dependence (Jiang et al., 2026).

Trust accounted for only part of the associations between cognitive appraisals and AI dependence. Both perceived empowerment and perceived threat remained directly associated with dependence after trust was included in the model. Students may continue to rely on AI because it reduces task demands, provides immediate feedback, or improves efficiency, even when their trust in the system is not particularly high (Henderson et al., 2026). Similarly, students who perceive greater threat may report less task delegation, engage in more careful verification, or rely on AI more cautiously (Wang G. et al., 2026). AI dependence may therefore involve both a trust-related pathway and a more direct appraisal-related pathway (Theoharakis and Mylonopoulos, 2026). These findings also have important implications for how AI-supported learning should be designed and implemented in educational contexts. From an educational perspective, the findings also highlight the importance of maintaining appropriate boundaries between AI assistance and cognitive responsibility. GenAI should function as a scaffold that supports students' cognitive engagement rather than replacing learners' responsibility for judgment and decision-making. Similar to teacher scaffolding, effective AI-supported learning requires students to actively evaluate AI outputs, retain ownership of learning processes, and use AI as a supportive resource rather than a substitute for independent thinking (Li and Wang, 2026). It should not be reduced to a simple outcome of technological trust, but may instead be understood as a reliance tendency associated with both appraisals of AI and judgments of its trustworthiness (Alarcon and Capiola, 2025).

The supplementary exploratory analysis showed numerical variation in the group-specific coefficients by GenAI usage frequency. For example, the association between trust and GenAI dependence increased numerically from the low-frequency group to the high-frequency group, whereas the direct empowerment-to-dependence coefficient decreased. These patterns are reported to preserve descriptive information, not as evidence of moderation or reliable group differences. Because measurement invariance was not tested and the low-frequency subgroup was small, the estimates cannot establish whether the constructs or structural paths differ across usage-frequency groups. Larger samples and formal invariance tests are needed before such numerical patterns can be interpreted as evidence of group differences (Polyportis, 2024).

## 6 Limitations and future research

This study has several limitations. First, the cross-sectional design allowed us to identify patterns of association among perceived AI empowerment, perceived AI threat, trust in AI, and AI dependence, but it did not establish temporal order or causality. Future research could use longitudinal, cross-lagged, or experimental designs to examine how AI appraisals, trust, and dependence develop over time and whether the proposed relationships reflect causal processes.

Second, the study relied on a convenience sample of Chinese university students, with participants concentrated in particular age and year-level groups. In addition, participants' academic majors or disciplinary backgrounds were not collected in the original survey, which limits our ability to determine whether the observed associations generalize across different fields of study. These sampling and contextual limitations constrain the generalizability of the findings. Future research should use larger and more diverse samples across countries, cultural settings, institutional types, and academic disciplines, and should systematically record participants' disciplinary backgrounds to assess the contextual stability of the model. In addition, all variables were measured through self-reports collected at a single time point. Although the measurement-model comparison did not support a single-factor account of the data, common method bias and self-report error cannot be ruled out. Future studies could combine survey responses with behavioral measures, learning-platform records, experimental task performance, or multi-source data.

Third, the study focused mainly on individual appraisals and trust, without systematically examining contextual conditions or individual differences. Future research could consider disciplinary background, task type, AI literacy, learning motivation, academic pressure, instructor expectations, and institutional AI policies. These factors may shape when and how trust is associated with GenAI dependence. Task risk may be particularly important, because reliance on AI may have different implications in low-stakes activities than in tasks involving assessment, academic integrity, or consequential decision making.

Finally, the multi-group analysis did not identify significant differences in structural paths across AI usage-frequency groups. The group-specific coefficients should therefore be treated as exploratory rather than as evidence of moderation. Future research should use larger and more evenly distributed samples, together with longitudinal designs or continuous moderation analyses, to test whether AI-use experience alters the relationships among appraisal, trust, and dependence. It is also important to distinguish usage frequency, habitual use, and GenAI dependence. Previous educational technology research has linked habit to both technology-use intention and actual use (Liu et al., 2025), but it remains unclear whether frequent or habitual AI use develops into cognitive or behavioral dependence and what role trust plays in that process. Future studies should further distinguish functional reliance, appropriate reliance, and potentially excessive dependence to determine whether greater trust supports effective human–AI collaboration or increases the risk of inappropriate reliance.

## 7 Conclusions

This study examined the relationships among perceived AI empowerment, perceived AI threat, trust in AI, and AI dependence among university students. Perceived AI empowerment was positively associated with both trust in AI and AI dependence, whereas perceived AI threat was negatively associated with both outcomes. Trust in AI was also positively associated with AI dependence and partially accounted for the associations between the two appraisals and dependence.

These findings indicate that students' dependence on GenAI is related not only to positive evaluations of its functions and practical value, but also to concerns about autonomy, learning ability, and potential risks. Students may view GenAI simultaneously as a resource that strengthens learning and as a source of possible negative consequences. These relatively independent appraisals are associated with trust and dependence in different directions. Overall, understanding university students' GenAI dependence requires attention to perceived empowerment, perceived threat, and judgments of AI reliability, rather than relying only on usage frequency, convenience, or general technology acceptance.

## Data Availability

The original contributions presented in the study are included in the article/Supplementary material, further inquiries can be directed to the corresponding author.
