# Supplementary material for: Perceived AI empowerment and threat in relation to university students' dependence on GenAI: the indirect role of trust in AI
Source: Front Psychol. 2026 Sep 23;17:1940317. doi: 10.3389/fpsyg.2026.1940317 (PMC13642636; doi:10.3389/fpsyg.2026.1940317)
Supplement: Supplementary file 1 [file Table_1.DOCX]

**Supplementary Material**

Questionnaire on University Students’ Perceptions and Use of GenAI

**1. Participant information and survey instructions**

Dear user,

Greetings! Thank you very much for taking the time to participate in this survey. Please read each question carefully and respond based on your actual situation and immediate impression. This questionnaire aims to understand students' perceptions, experiences, and behaviors related to AI tool usage.

您好！感谢您抽出宝贵时间参与本次问卷调查。请您认真阅读每一项问题，并根据您的实际情况和第一反应进行作答。本问卷旨在了解学生对AI工具使用的感知、体验以及使用行为。

Participation in this survey is entirely voluntary and anonymous. The data collected will be used solely for academic research purposes. The survey does not involve personal privacy and will not negatively impact your physical or mental health. If you feel uncomfortable while answering, you may stop at any time.

本调查为完全自愿参与，采取匿名形式，所收集的数据仅用于学术研究，不涉及您的个人隐私，也不会对您的身心健康产生不良影响。如您在填写过程中感到不适，可以随时终止问卷作答。

**2. Translation and adaptation procedure**

A. Your gender is：你的性别是：

1) Male 男	2) Female 女

B. Your age：你的年龄：

1) <22 years old	2) 22–25 years old	3) 26–30 years old	4) ≥30 years old

C. Your academic year：你的年级是：

1) Freshman 大一	2) Sophomore 大二	3) Junior 大三	4) Senior or above大四及以上

D. GenAI usage frequency：GenAI使用频率：

1) Few times (≤1 time per month) 很少（一个月≤1次）

2) Sometimes (1–3 times per week) 有时（一周1–3次）

3) Frequently (≥4 times per week) 频繁（一周≥4次）

**3. English and Chinese versions of the measurement items**

Table S1. English and Chinese versions of the measurement items

| **Construct** | **English item** | **Chinese item** | **Source(s)** |
| --- | --- | --- | --- |
| Perceived  AI Empowerment | PE1 After using GenAI, I feel that I am more capable of completing tasks. | PE1 使用GenAI后，我觉得自己更有能力完成任务。 | (Kong & Yang, 2025; Kong et al., 2025; Spreitzer, 1995) |
|  | PE2 Using GenAI enables me to solve problems more efficiently. | PE2 使用GenAI后，我能够更高效地解决问题。 |  |
|  | PE3 Using GenAI gives me a greater sense of control when completing tasks. | PE3 使用GenAI后，我在完成任务时更有掌控感。 |  |
|  | PE4 Using GenAI enables me to produce more valuable outcomes. | PE4 使用GenAI后，我能够产出更有价值的结果。 |  |
|  | PE5 With GenAI, I am more confident in completing complex tasks. | PE5 使用GenAI后，我更有信心完成复杂任务。 |  |
|  | PE6 Using GenAI makes it easier for me to find effective ways to complete tasks. | PE6 使用GenAI后，我更容易找到完成任务的有效方法。 |  |
|  | PE7 I feel that GenAI enables me to handle more demanding tasks. | PE7 使用GenAI后，我觉得自己能够应对更高要求的任务。 |  |
| Perceived  AI  Threat | PT1 I am concerned that using GenAI may lead to unpredictable negative consequences. | PT1 我担心使用GenAI可能带来不可预期的负面后果。 | (Kieslich et al., 2021; Wang & Wang, 2022) |
|  | PT2 I am concerned that the development of GenAI may weaken my autonomy or value in performing tasks. | PT2 我担心GenAI的发展会削弱我在任务中的主动性或价值。 |  |
|  | PT3 I am concerned that GenAI may replace human roles in certain situations. | PT3 我担心GenAI在某些情况下可能会取代人的作用。 |  |
|  | PT4 I believe that overreliance on GenAI may bring certain risks or threats. | PT4 我认为过度依赖GenAI会带来一定的风险或威胁。 |  |
|  | PT5 I am concerned that long-term use of GenAI may weaken my ability to think independently. | PT5 我担心长期使用GenAI会削弱我独立思考的能力。 |  |
| Trust in AI | TR1 I believe that GenAI can provide valuable support in most situations. | TR1 我相信GenAI在多数情况下能够提供有价值的支持。 | (Jian et al., 2000) |
|  | TR2 I believe that the information provided by GenAI is generally accurate. | TR2 我认为GenAI提供的信息通常是准确的。 |  |
|  | TR3 I usually have confidence in the suggestions or results provided by GenAI. | TR3 我通常对GenAI的建议或结果有信心。 |  |
|  | TR4 I believe that GenAI can provide reliable assistance when performing tasks. | TR4 我认为GenAI能够在任务中提供稳定的帮助。 |  |
|  | TR5 I believe that the suggestions provided by GenAI are generally reasonable. | TR5 我认为GenAI给出的建议通常具有一定的合理性。 |  |
|  | TR6 I am willing to use information provided by GenAI as a reference for decision-making. | TR6 我愿意把GenAI提供的信息作为决策参考。 |  |
| AI Dependence | DE1 I have developed a habit of using GenAI to assist me in completing tasks. | DE1 我习惯在完成任务时使用GenAI辅助自己。 | (Morales-García et al., 2024; Wu et al., 2026) |
|  | DE2 I feel that completing tasks would become more difficult without GenAI. | DE2 我觉得不使用GenAI完成任务会变得更困难。 |  |
|  | DE3 In most situations, I would consider using GenAI first to help me complete tasks. | DE3 在多数情况下，我会优先考虑使用GenAI来帮助完成任务。 |  |
|  | DE4 Even when I am capable of completing a task independently, I tend to use GenAI first. | DE4 即使我能够独立完成任务，我仍倾向于优先使用GenAI。 |  |
|  | DE5 For some tasks, GenAI has become an indispensable tool for me. | DE5 我在完成某些任务时，GenAI是不可或缺的工具。 |  |
|  | DE6 When completing certain tasks without GenAI, I feel noticeably less confident or secure. | DE6 我完成某些任务时不使用GenAI会明显缺乏安全感。 |  |
|  | DE7 I have become accustomed to using GenAI to verify whether my ideas are correct. | DE7 我习惯通过GenAI来确认自己的想法是否正确。 |  |

**Translation and adaptation procedure.** The Chinese-language questionnaire was administered to participants. The measurement items were adapted from previously validated English-language instruments and contextualized for university students’ use of GenAI. The adapted items were translated into Chinese and checked against the English versions for semantic equivalence, conceptual consistency, and readability. Minor wording adjustments were made where necessary to improve contextual appropriateness without changing the intended meaning of the constructs. The complete English and Chinese versions are provided below.

**4. Sources and adaptation of the measurement items**

Table S2. Source items, adapted items, and adaptation rationale

| **Construct** | **Source item(s) / conceptual basis** | **Adapted item used in this study** | **Adaptation rationale** |
| --- | --- | --- | --- |
| Perceived  AI Empowerment | Item 5: “I can learn to be better at solving problems with AI.” Item 9: “I have confidence in my ability to solve problems with AI.” | After using GenAI, I feel that I am more capable of completing tasks. | The original wording was contextually adapted to university students’ use of GenAI in learning and task-completion contexts while preserving the underlying meaning of perceived competence, effectiveness, and capability enhancement.  (Kong & Yang, 2025; Kong et al., 2025; Spreitzer, 1995) |
|  | Item 2: “I want to solve problems with AI to make people’s lives better.” Item 3: “I want to solve problems with AI to make daily life easier.” | Using GenAI enables me to solve problems more efficiently. |  |
|  | Item 1: “I want to apply my AI knowledge and skills to solve problems in daily life.” Item 2: “I want to solve problems with AI to make people’s lives better.” | Using GenAI gives me a greater sense of control when completing tasks. |  |
|  | Item 13: “Using AI to solve problems will help me achieve my goals.” Item 15: “Using AI to solve problems is important to me.” Item 16: “Using AI to solve problems is useful to me.” | Using GenAI enables me to produce more valuable outcomes. |  |
|  | Item 7: “I think of myself as someone who can solve problems with AI.” Item 8: “I have the knowledge and skills to solve problems with AI.” Item 9: “I have confidence in my ability to solve problems with AI.” | With GenAI, I am more confident in completing complex tasks. |  |
|  | Item 5: “I can learn to be better at solving problems with AI.” Item 8: “I have the knowledge and skills to solve problems with AI.” | Using GenAI makes it easier for me to find effective ways to complete tasks. |  |
|  | Item 8: “I have the knowledge and skills to solve problems with AI.” Item 10: “I can think creatively when I use AI to solve problems.” | I feel that GenAI enables me to handle more demanding tasks. |  |
| Perceived  AI  Threat | Q46：“I am afraid of various problems potentially associated with an AI technique/product.” | I am concerned that using GenAI may lead to unpredictable negative consequences. | The original wording was adapted to the GenAI-supported learning context by replacing general or domain-specific AI references with situations relevant to university students’ learning and task-related activities, while retaining the underlying meaning of perceived risk and potential negative consequences. (Kieslich et al., 2021; Wang & Wang, 2022) |
|  | Q48：“I am afraid that an AI technique/product may lead us to lose our autonomy.” | I am concerned that the development of GenAI may weaken my autonomy or value in performing tasks. |  |
|  | Q18：“I am afraid that an AI technique/product may replace humans.” | I am concerned that AI may replace human roles in certain situations. |  |
|  | Q16：“I am afraid that an AI technique/product may make us dependent.” | I believe that overreliance on GenAI may bring certain risks or threats. |  |
|  | Q11：“I am afraid that if I begin to use AI techniques/products I will become dependent upon them and lose some of my reasoning skills.” | I am concerned that long-term use of GenAI may weaken my ability to think independently. |  |
| Trust in AI | I trust the system. | I believe that GenAI can provide valuable support in most situations. | The original items were contextually adapted from general automation settings to the GenAI context, with wording revised to reflect students’ evaluations of GenAI competence, reliability, accuracy, and supportive value.  (Jian et al., 2000) |
|  | The system is reliable. | I believe that the information provided by GenAI is generally accurate. |  |
|  | I am confident in the system. | I usually have confidence in the suggestions or results provided by GenAI. |  |
|  | I can rely on the system. | I believe that GenAI can provide reliable assistance when performing tasks. |  |
|  | The system behaves in a consistent manner. | I believe that the suggestions provided by GenAI are generally reasonable. |  |
|  | I rely on the system when making decisions | I am willing to use information provided by GenAI as a reference for decision-making. |  |
| AI Dependence | Item 4: “I've grown accustomed to completing academic tasks with AI; being without it would make me feel unsettled.” | I have developed a habit of using GenAI to assist me in completing tasks. | The original items were adapted to university students’ use of GenAI by contextualizing the wording around task completion, problem solving, and other learning-related activities while retaining the underlying meaning of reliance on AI. (Morales-García et al., 2024; Wu et al., 2026) |
|  | Item 8: “Without the help of AI, I find it difficult to complete my assignments efficiently.” | I feel that completing tasks would become more difficult without GenAI. |  |
|  | Item 5: “Whenever I encounter a difficult problem, my first instinct is to hope AI can help me; otherwise, I feel tense.” | In most situations, I would consider using GenAI first to help me complete tasks. |  |
|  | Item 25: “I am capable of completing tasks without AI, but I still cannot resist using it.” | Even when I am capable of completing a task independently, I tend to use GenAI first. |  |
|  | Item 10: “I can no longer manage complex academic tasks without the assistance of AI.” | For some tasks, GenAI has become an indispensable tool for me. |  |
|  | Item 13: “I feel inadequate in my studies without the support of AI.” | When completing certain tasks without GenAI, I feel noticeably less confident or secure. |  |
|  | Item 18: “I am accustomed to letting AI make judgments and decisions for me rather than fully trusting my own judgment.” | I have become accustomed to using GenAI to verify whether my ideas are correct. |  |

**References**

Jian, J. Y., Bisantz, A. M., & Drury, C. G. (2000). Foundations for an empirically determined scale of trust in automated systems. *International Journal of Cognitive Ergonomics*, *4*(1), 53–71. <https://doi.org/10.1207/S15327566IJCE0401_04>

Kieslich, K., Lünich, M., & Marcinkowski, F. (2021). The Threats of Artificial Intelligence Scale (TAI). *International Journal of Social Robotics*, *13*(7), 1563–1577. <https://doi.org/10.1007/s12369-020-00734-w>

Kong, S. C., & Yang, Y. (2025). Developing and validating an artificial intelligent empowerment instrument: evaluating the impact of an artificial intelligent literacy programme for secondary school and university students. *Research and Practice in Technology Enhanced Learning*, *20*, 024. <https://doi.org/10.58459/rptel.2025.20024>

Kong, S. C., Zhu, J., & Yang, Y. N. (2025). Developing and validating a scale of empowerment in using artificial intelligence for problem-solving for senior secondary and university students. *Computers and Education: Artificial Intelligence*, *8*, 100359. <https://doi.org/10.1016/j.caeai.2024.100359>

Morales-García, W. C., Sairitupa-Sanchez, L. Z., Morales-García, S. B., & Morales-García, M. (2024). Development and validation of a scale for dependence on artificial intelligence in university students. *Frontiers in Education*, *Volume 9 - 2024*. <https://doi.org/10.3389/feduc.2024.1323898>

Spreitzer, G. M. (1995). Psychological Empowerment in the Workplace: Dimensions, Measurement, and Validation. *The Academy of Management Journal*, *38*(5), 1442–1465. <https://doi.org/10.2307/256865>

Wang, Y. Y., & Wang, Y. S. (2022). Development and validation of an artificial intelligence anxiety scale: an initial application in predicting motivated learning behavior. *Interactive Learning Environments*, *30*(4), 619–634. <https://doi.org/10.1080/10494820.2019.1674887>

Wu, H., Ni, H., Luo, W., & Wu, T. (2026). Development and validation of the AI dependence scale for Chinese undergraduates and a preliminary exploration. *Frontiers in Psychology*, *Volume 16 - 2025*. <https://doi.org/10.3389/fpsyg.2025.1725393>
